# A Major Role for Side-Chain Polyglutamine Hydrogen Bonding in Irreversible Ataxin-3 Aggregation

**DOI:** 10.1371/journal.pone.0018789

**Published:** 2011-04-13

**Authors:** Antonino Natalello, Anna Maria Frana, Annalisa Relini, Alessandra Apicella, Gaetano Invernizzi, Carlo Casari, Alessandra Gliozzi, Silvia Maria Doglia, Paolo Tortora, Maria Elena Regonesi

**Affiliations:** 1 Department of Biotechnologies and Biosciences, University of Milano-Bicocca, Milan, Italy; 2 Department of Physics, University of Genoa, Genoa, Italy; 3 Energy Department, Polythechnic of Milan, Milan, Italy; Monash University, Australia

## Abstract

The protein ataxin-3 consists of an N-terminal globular Josephin domain (JD) and an unstructured C-terminal region containing a stretch of consecutive glutamines that triggers the neurodegenerative disorder spinocerebellar ataxia type 3, when it is expanded beyond a critical threshold. The disease results from misfolding and aggregation, although the pathway and structure of the aggregation intermediates are not fully understood. In order to provide insight into the mechanism of the process, we monitored the aggregation of a normal (AT3Q24) ataxin-3, an expanded (AT3Q55) ataxin-3, and the JD in isolation. We observed that all of them aggregated, although the latter did so at a much slower rate. Furthermore, the expanded AT3Q55 displayed a substantially different behavior with respect to the two other variants in that at the latest stages of the process it was the only one that did the following: i) lost its reactivity towards an anti-oligomer antibody, ii) generated SDS-insoluble aggregates, iii) gave rise to bundles of elongated fibrils, and iv) displayed two additional bands at 1604 and 1656 cm^−1^ in FTIR spectroscopy. Although these were previously observed in other aggregated polyglutamine proteins, no one has assigned them unambiguously, yet. By H/D exchange experiments we show for the first time that they can be ascribed to glutamine side-chain hydrogen bonding, which is therefore the hallmark of irreversibly SDS-insoluble aggregated protein. FTIR spectra also showed that main-chain intermolecular hydrogen bonding preceded that of glutamine side-chains, which suggests that the former favors the latter by reorganizing backbone geometry.

## Introduction

Ataxin-3 (AT3) is a 42 kDa intracellular protein that is responsible for the polyglutamine (polyQ) disease spinocerebellar ataxia type 3, when the length of its polyQ tract exceeds about 50 consecutive residues [1]–[3]. It consists of the N-terminal Josephin domain (JD), whose structure has recently been solved by NMR [4] and of a disordered C-terminal domain, wherein the polyQ stretch is close to the C-terminus [5]. The JD is structurally similar to some members of the cysteine protease family, and also has the catalytic triad found in these enzymes [4], sustaining ubiquitin hydrolase activity in AT3 [6], [7].

Plenty of work has been carried out in recent years to elucidate the mechanisms underlying the aggregation of AT3 and other polyQ-containing proteins, which has highlighted the complexity of the process, showing in particular that the protein context and the polyQ-flanking regions critically affect the mode and outcome of aggregation [8], [9]. As far as AT3 is concerned, it has been shown that the JD also has an intrinsic amyloidogenic potential, which results in the wild-type protein's capability to aggregate, a feature also shared by truncated variants, including the JD in isolation [10], [11]. This implies that the aggregation pathway consists of two steps: the first, only requiring the JD, gives rise to SDS-soluble protofibrils, whereas the second is solely accessible to variants carrying expanded polyQs and results in the formation of mature, SDS-insoluble fibrils [12], [13]. Nevertheless, these variants display the fastest aggregation kinetics, which suggests that the polyQ tract also affects the mode of JD aggregation [14]. It is noteworthy that other proteins, in particular ataxin-1 and huntingtin exon-1, also have polyQ-flanking regions that participate in the process and substantially affect the features of the resulting aggregates [15], [16]. Interestingly, Robertson and coworkers recently showed that the small heat-shock protein alphaB-crystallin retards or prevents aggregation of both JD and AT3 by selectively interacting with an extensive region of the JD [17]. This finding further highlights the key role of the latter in the first step of the aggregation process.

Based on x-ray diagrams of a short polyQ peptide (D_2_Q_15_K_2_) and on theoretical considerations, Perutz and coworkers proposed that amyloid fibers generated by expanded polyQs would consist of at least two β-helical turns each containing 20 residues, and that residues in successive turns would be linked by hydrogen bonds between both main chain and side chain amides [18]. Although Sikorski and Atkins suggested an alternative model wherein the peptide adopts a hairpin conformation, they also surmise that the glutamine residues are hydrogen bonded via both main- and side-chain amides [19]. Nevertheless, experimental data available to date do not adequately depict the changes taking place at the molecular level at different stages of aggregation in both normal and expanded polyQs. In particular, as far as the latter are concerned, the molecular events that lead to the conversion of reversibly aggregated oligomers into stably aggregated fibrils are still largely obscure.

Taking advantage of several analytical methods, especially FTIR spectroscopy, we provide here an in-depth analysis of such changes in a normal and an expanded AT3 variant, as well as in the JD in isolation. In particular, our results provide the first experimental evidence that side-chain hydrogen bonding of polyQs is responsible for irreversible aggregation and SDS-insolubility of the expanded form.

## Results

### Different truncated, normal full length and expanded AT3 variants undergo aggregation as detected by Thioflavin T (ThT) fluorimetry

To achieve a deeper understanding of the structural changes associated with AT3 fibrillogenesis, we investigated different forms of AT3, i.e.: AT3/182Δ (truncated at the residue 182 and consisting of the sole JD); AT3Q24 and AT3Q55 (full-length normal and pathogenic, carrying 24 and 55 consecutive glutamines, respectively).

**Figure 1:**
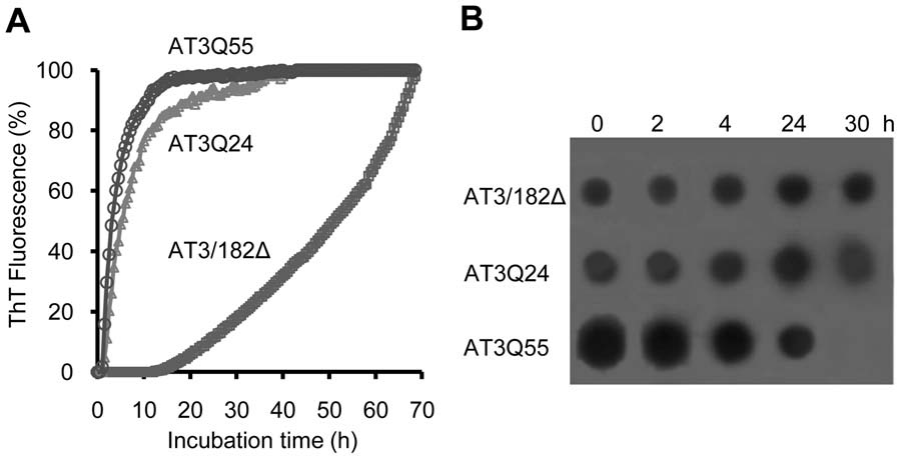
Progress of the aggregation of AT3 variants. (A) Aggregation was monitored by measuring changes in ThT fluorescence of proteins incubated at 37°C at a 25 µM concentration in PBS, pH 7.2, and in the presence of 20 µM ThT. Fluorescence was recorded using a plate reader with values read every 30 min. Individual values are the mean of three independent determinations, with standard deviations never exceeding 5% of the mean value. Fluorescence is relative to the highest value, achieved at a 60 h-incubation. (B) Time course of oligomeric AT3 form appearance, as detected by dot blot. AT3 variants were incubated at 37°C in PBS, pH 7.2, for the times indicated. Samples were withdrawn, applied to an Immobilon membrane, immunodecorated using anti-oligomer, conformation-specific antibodies (19) and revealed using ECL Western blotting reagent.

By ThT fluorimetry, we first assessed the propensity of the different variants to aggregate at 37°C over a time span of 68 h, which showed that all of them were capable of doing so, although at different rates (Fig. 1A). As a control, β-lactoglobulin did not undergo any fluorescence increase under the same conditions (data not shown). The AT3/182Δ variant also underwent aggregation as expected [11], although at a much slower rate than the others. The polyQ-carrying variants, AT3Q24 and AT3Q55, aggregated much more rapidly than the truncated ones, AT3Q55 displaying a somewhat higher rate than AT3Q24. In particular, they attained a plateau at about 10 h and 30 h, respectively. No lag phase was detectable for these two variants. This, however, might be due to the high rate of the process.

### Dot-blot assays show a different aggregation pattern of AT3Q55 compared to the other variants

We also analyzed the aggregation products generated by the different AT3 variants by assessing the appearance of oligomeric forms in dot-blot assays performed at different incubation times. To this end, we used an antibody that is capable of selectively recognizing the earliest aggregation products but not mature fibrils [20]. We thus observed that the AT3Q55 oligomers progressively decreased from the beginning and disappeared completely at 30 h, whereas the two other variants displayed an approximately constant level of oligomers over the entire incubation time (Fig. 1B). This is strongly suggestive of the unique capability of the expanded form to evolve towards mature fibrils, as also confirmed by FTIR spectroscopy, filter trap assays and AFM (reported below). The reactivity detected at zero-time suggests that oligomeric forms are present from the very beginning of the incubation. It should be stressed, however, that the initial amount of aggregated protein, quantified on the basis of FTIR bands at 1630–1624 and 1695 cm^−1^ as outlined below, was hardly detectable in all AT3 variants.

### The secondary structures of AT3 variants are identified by FTIR spectroscopy

**Figure 2:**
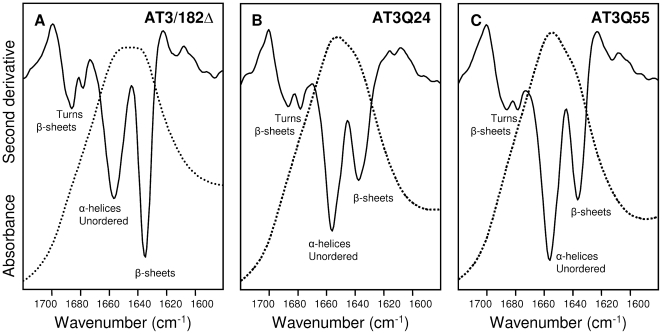
FTIR spectra of freshly purified AT3 variants. Absorption spectra (dotted line) and their second derivatives (continuous line) in the amide I region of AT3 variants: JD (A), AT3Q24 (B), and AT3Q55 (C). Band assignment to the secondary structure components is indicated.

FTIR spectroscopy can very precisely discriminate between closely related but different protein secondary structures for instance, β-intermolecular *versus* β-intramolecular sheets [21], which is relevant to the subject of the present work. We thus monitored the time-dependent structural changes of the different AT3 variants under investigation. However, we first assessed the secondary structure content of freshly purified proteins. In Fig. 2A, the absorption spectrum of the JD in the amide I band region is presented. This is mainly contributed by the C = O peptide bond absorption whose peak position is sensitive to the protein's secondary structure [22]–[25]. In order to resolve this band into its overlapping components, the second derivative analysis of the spectrum [22] was performed. Two main components, appearing as negative peaks, were detected at 1657 and 1635 cm^−1^. The former can be assigned to α-helices and random coils; the latter, along with a shoulder around 1690 cm^−1^, to native, intramolecular β-sheets. In addition, two low-intensity components at about 1688 and 1678 cm^−1^ were found in the typical absorption region of β-turns [22]–[25], in keeping with the secondary structure components reported for the JD [4], [11]. The second derivative spectra of the two other AT3 variants were similar to each other and displayed the same secondary structure components found in the JD (Fig. 2B, 2C). They, however, exhibited a 1657 cm^−1^ band, which was more pronounced than that around 1635 cm^−1^, contrary to the JD (Fig. 2A). This indicates that the C-terminal disordered domain must contribute to the absorption of the 1657 cm^−1^ component. Thus, we conclude that the secondary structure of this stretch is mainly α-helical and/or disordered, consistent with CD spectra and computational studies [11]. It is also noteworthy that the glutamine side-chain infrared response, which in the amide I region is expected in the ranges 1687–1668 cm^−1^ and 1611–1586 cm^−1^ [26], was undetectable in the freshly purified AT3Q55 spectrum, probably due to structural heterogeneity of the polyQ stretch (Fig. 2B, 2C).

### FTIR spectroscopy highlights major changes in the secondary structure of AT3 variants upon incubation under physiological conditions

**Figure 3:**
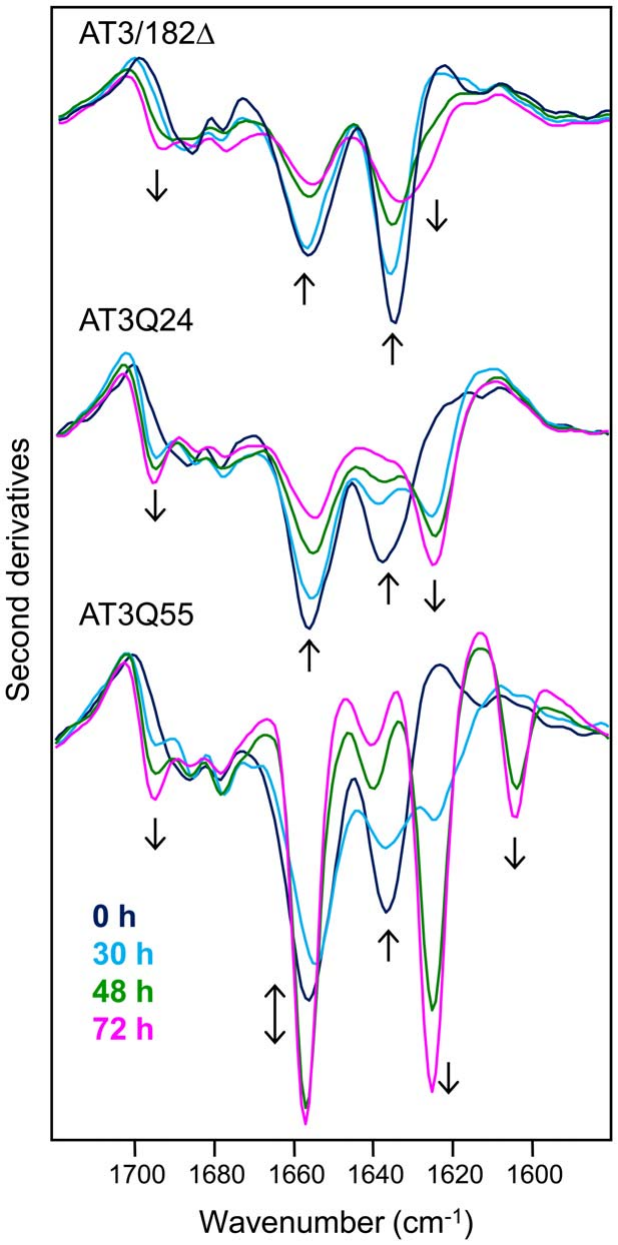
Kinetics of aggregation of AT3 variants monitored by FTIR spectroscopy. Second derivative spectra of AT3 variants were taken at different times of incubation in PBS at 37°C. Arrows point to increasing time.

Previous studies showed that AT3 undergoes aggregation under physiological conditions, with resulting generation of amyloid-like structures [13], [14]. To assess the changes in secondary structure associated with this process, we incubated the AT3 variants under investigation at 37°C, and collected FTIR spectra at different times (Fig. 3). JD second derivative spectra showed a slow decrease in the 1657 cm^−1^ and 1635 cm^−1^ components, which are contributed by α-helix/disordered and native β-sheet structures, respectively. In addition, between 48 and 72 h of incubation, two further low-intensity components appeared as shoulders around 1630 cm^−1^ and 1694 cm^−1^. These bands, which became better resolved at 168 h (Fig. S1), occur in the absorption region of intermolecular β-sheets, so they are diagnostic of aggregation [21], [27], [28]. A similar behavior was observed in the case of AT3Q24, with two major differences. First, the peak position of the aggregation band at 1630 cm^−1^ in the case of the JD, occurred instead around 1624 cm^−1^ (Fig. S1). This downshift points to tighter aggregates in AT3Q24, probably resulting from stronger hydrogen bonding and/or increased number of β-strands in the intermolecular β-sheets ([29] and references therein). Second, the aggregation of this variant was distinctly faster than that of the JD, as apparent from the comparison of the aggregation bands of the different proteins (shown in Fig. 3). This result fits well with the kinetics monitored by ThT fluorescence (Fig. 1A), thus confirming that the disordered C-terminal domain significantly enhances the aggregation rate. Furthermore, these spectral data also show that in the presence of this domain, the resulting aggregation products are to some extent structurally different from those generated by the JD alone.

The time-dependent spectral changes in the second derivative spectra of the pathogenic AT3Q55, whilst sharing some features with those of the other forms, also displayed unique traits. In particular, the native β-sheet component at 1635 cm^−1^ underwent a decrease in intensity like the other forms, whereas the α-helix/disordered component at 1657 cm^−1^ first decreased until 30 h. Thereafter it increased again, thus exceeding the zero-time intensity (Fig. 3). The absorption spectrum of AT3Q55 displayed a band at 1657 cm^−1^ whose intensity was lower in the aggregated protein compared to the freshly prepared sample (Fig. S2). This apparently different behavior of relative band intensities in the absorption and second derivative spectra can be accounted for if one considers that peak intensities in the second derivative spectra are inversely correlated with band widths [22], with resulting intensification of narrow bands. The observed narrowing of the 1657 cm^−1^ component is suggestive of a highly ordered structure.

Furthermore, the aggregation bands around 1624 cm^−1^ and 1695 cm^−1^ started to increase from the earliest stages of the incubation, reaching intensities higher than those of the other variants (Fig. 3). This holds true in particular for the 1624 cm^−1^ band intensity that in AT3Q55 aggregates was twice as strong after a 168-h incubation as that of the AT3Q24 aggregates (Fig. S1). Most notably, we also observed an additional band at 1604 cm^−1^ (Fig. 3) that was also retained in the spectrum of AT3Q55 mature aggregates (Fig. S1). Although it was already found in protein and peptide aggregates containing polyQs [16], [30]–[32], it has not yet been unambiguously assigned.

### H/D exchange experiments allow one to unambiguously identify the IR spectral components contributed by irreversibly aggregated, expanded polyQ stretches

**Figure 4:**
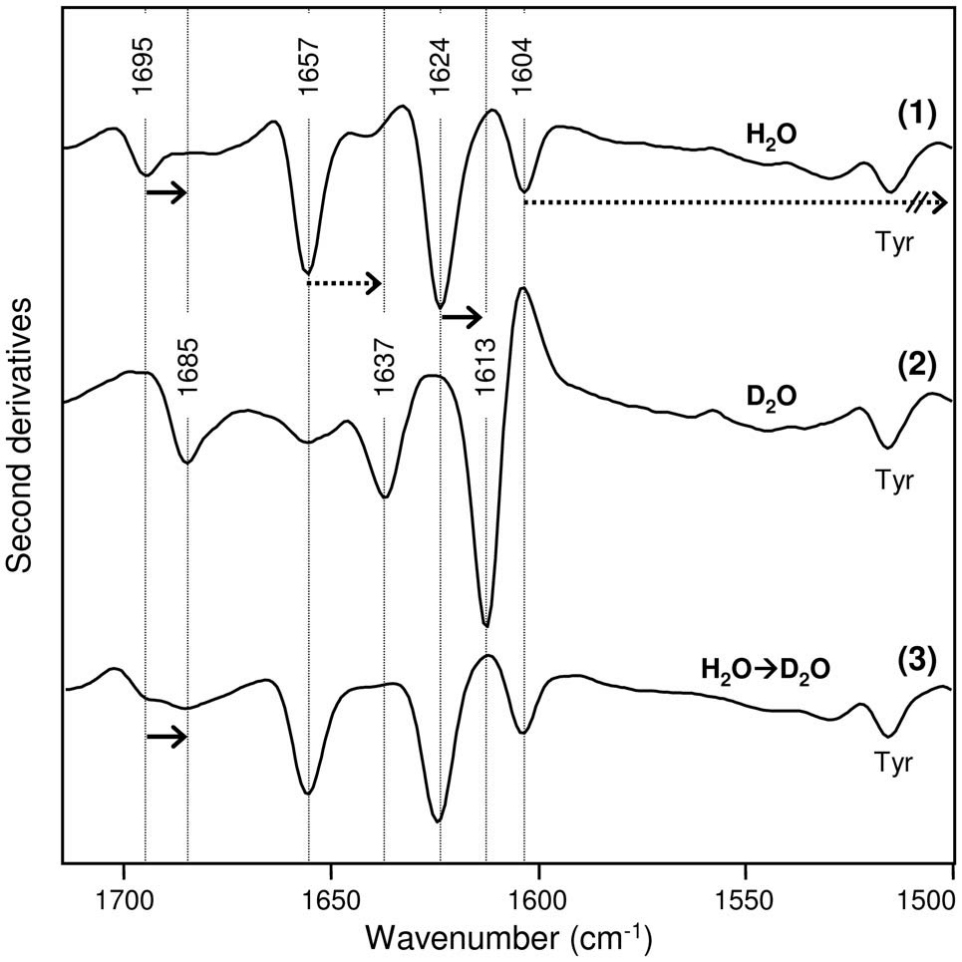
Effect of H/D exchange on FTIR spectra of ATQ55 aggregates. Freshly purified AT3Q55 was incubated in H_2_O/PBS (spectrum 1) and in D_2_O/PBS (spectrum 2) at 37°C for 168 h. The second derivative spectra of the pelletted aggregates are reported. Upon deuteration, the shift of the cross β-sheet bands (continuous arrows) and the glutamine side chain bands (dotted arrows) are indicated. In a different H/D exchange experiment, freshly purified AT3Q55 was incubated in H_2_O/PBS at 37°C for 168 h and the obtained mature aggregates were subsequently submitted to three cycles of lyophilization and resuspension in D_2_O, as described in Materials and Methods. Then, the mature aggregates were resuspended in D_2_O and their ATR/FTIR second derivative spectrum recorded (spectrum 3). No appreciable H/D exchange with solvent took places under these conditions, except for the minor one of the 1695 cm^−1^ component.

The amide I and II second derivative spectrum of the AT3Q55 aggregates, collected by centrifugation of the protein solution after 168 h of incubation, is presented in Fig. 4.

In H_2_O, the amide I spectrum displayed four well resolved bands at 1695 cm^−1^, 1657 cm^−1^, 1624 cm-1 and 1604 cm^−1^. Whereas the 1695 cm^−1^ and 1624 cm^−1^ components can be definitely assigned to the intermolecular β-sheet interaction of the protein aggregates [21], [27], [28], the two other bands were assigned by other authors to different vibrational modes. In particular, bands at about 1658 cm^−1^ and 1604 cm^−1^ were found in the spectrum of model polyQ peptides and both assigned to intermolecular β-sheets [30]. Such bands were also observed in the amyloid aggregate spectra of the cyclin-dependent kinase subunit by Bader et al. [31], who suggested an assignment to hydrogen-bonded glutamine side-chains instead. Thus, for a conclusive assignment of these bands, we performed H/D exchange experiments.

Indeed, several reports show that in both free and protein-bound glutamines, the C = O stretching absorption of side chains was found to downshift from 1687–1668 cm^−1^ in H_2_O to 1654–1635 cm^−1^ in D_2_O, while the NH_2_ deformation mode of side chains underwent a very large shift, from 1611–1586 cm^−1^ in H_2_O to about 1163 cm^−1^ in D_2_O (for a review see ref. [26]). These downshifts, which are considerably larger than those expected for the protein backbone bands upon deuteration [26], [33], allowed us to discriminate between glutamine side-chain IR response and that of protein backbone in aggregates. We therefore incubated freshly prepared AT3Q55 at 37°C in D_2_O. Under these conditions, deuterated aggregates were obtained, as indicated by the downshifted IR band positions in the amide I region (Fig. 4). Actually, in D_2_O, the two bands at 1695 cm^−1^ and 1624 cm^−1^ downshifted to 1685 cm^−1^ and 1613 cm^−1^, respectively, as expected for intermolecular β-sheet backbone modes. Most notably, the bands at 1657 cm^−1^ and 1604 cm^−1^ displayed major downshifts, the former to 1637 cm^−1^, the latter actually disappearing from the amide I and amide II regions and shifting into the crowded absorption region around 1160 cm^−1^. These results suggest that the 1657 cm^−1^ and 1604 cm^−1^ components can be assigned to the C = O stretching and NH_2_ deformation modes of the glutamine side chains, respectively, in AT3Q55 amyloid aggregates. To support this assignment, we performed an additional H/D exchange experiment aimed at further investigating glutamine interactions in the AT3Q55 amyloid aggregates. Aggregates obtained after incubation of the freshly purified protein in H_2_O buffer at 37°C for 168 h were subsequently submitted to three cycles of lyophilization and resuspension in D_2_O, as described in Materials and Methods. After this drastic treatment, the FTIR spectrum of AT3Q55 mature aggregates (Fig. 4) still displayed the two components at 1657 and at 1604 cm^−1^, thus showing that no H/D exchange with the solvent took place under these conditions. This makes it possible to exclude the assignment of the 1657 cm^−1^ band to unordered structures. This result is indeed expected for glutamines involved in strong side chain-side chain (and possibly side chain-backbone) hydrogen bonding.

The above experiments therefore provide conclusive evidence supporting the previous proposed models [18], [19], whereby glutamine residues in irreversibly aggregated, expanded polyQ stretches, are linked by hydrogen bonds between both main chain and side chain amides. It is worth mentioning that the C = O band of glutamine side chains in AT3Q55 amyloid aggregates occurs in H_2_O at 1657 cm^−1^, which is outside the typical range of free glutamine (1687–1668 cm^−1^). This downshift is further evidence that glutamine side chains are involved in strong hydrogen bonding.

### AT3Q55 intermolecular backbone hydrogen bond formation precedes that of glutamine side chains, the latter resulting in irreversible aggregation

**Figure 5:**
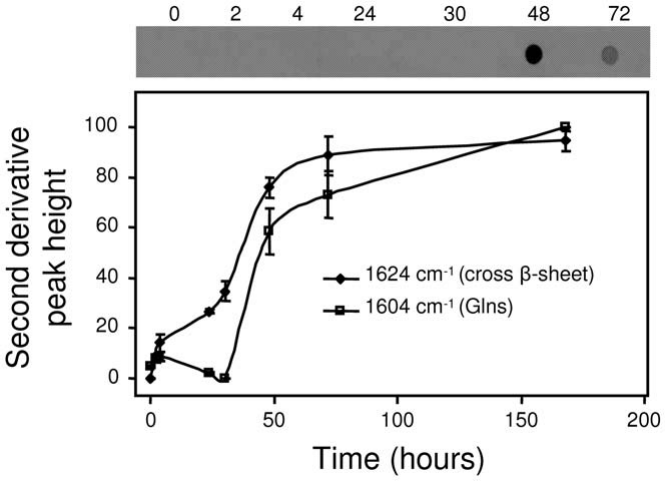
Time course of AT3Q55 aggregation upon incubation at 37°C in PBS. Aggregation was monitored by detecting either the appearance of SDS-insoluble aggregates by filter trap assays (upper panel), or cross β-sheet (1625 cm^−1^) and glutamine side chain bands (1604 cm^−1^) (lower panel). The band heights from the second derivative spectra (normalized at the tyrosine peak to allow for possible differences in protein content) are reported as percentage variations in the time span 0–168 h. Standard deviations from three independent experiments are indicated.

We also monitored the kinetics of appearance of the bands at 1624 cm^−1^ and 1604 cm^−1^ that are assigned to β-sheet intermolecular interactions and glutamine side chain NH_2_ deformation mode, respectively. The time course shows that the increase in the β-sheet intermolecular band started at the very beginning of the incubation, reaching a plateau after 50–80 h (Fig. 5). In contrast, the glutamine band appeared after 30 h and leveled off in a time interval comparable to that of β-sheets. Remarkably, the appearance of the glutamine band was paralleled by that of SDS-insoluble aggregates, as detected by filter trap assays (Fig. 5). This distinctive IR response could not be abolished by SDS treatment (Fig. S3). It is well known that only expanded AT3 variants undergo irreversible aggregation, whereas wild-type forms do not go beyond the first reversible aggregation step [13]. Thus, our results also demonstrate that the rearrangement of glutamine side chains and the resulting side-chain hydrogen bonding observed in the expanded variant is the key event resulting in the generation of irreversible aggregates.

### AFM confirms that only the expanded AT3Q55 generates fibrils

Tapping mode AFM was employed to investigate the morphologies of the aggregates formed by JD, AT3Q24 and AT3Q55 at fixed aggregation times. At all the times analyzed, the JD formed globular particles with a mean height of 3.6±0.1 nm that displayed a relatively low propensity to form clusters of spheroidal aggregates even after 72 h from the start of the aggregation experiment (Fig. S4). A more complex aggregation behavior was shown by AT3Q24 that evolved from globular particles with a mean height of 4.9±0.2 nm at t = 0 to clusters of particles at 24 h (Fig. S5A and B). After 72 h, most of the aggregates still exhibited a non-fibrillar morphology, while only few short protofibrils 250–300 nm long and about 4 nm high were observed (Fig. S5C).

**Figure 6:**
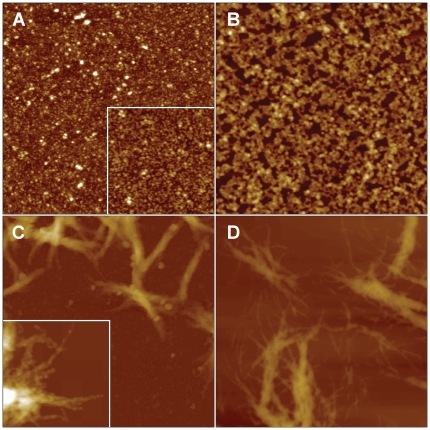
Tapping mode AFM images (height data) of the expanded variant AT3Q55 incubated at 37°C. The protein was incubated for: (A) 0 h (inset 2 h); (B) 24 h; (C) 48 h; (D) 72 h. The samples imaged in the inset of (C) and in (D) were treated with SDS (see Materials and Methods) to remove non fibrillar material. Scan size: (A, B, D) 2 µm; (C) 5 µm; insets 1 µm. Z range: (A) 10 nm, inset 20 nm; (B) 30 nm; (C) 700 nm, inset 180 nm; (D) 150 nm.

At the start of aggregation the expanded variant AT3Q55 also showed a distribution of globular particles of variable size (Fig. 6A), with a mean height of 3.1±0.2 nm. A globular morphology was also detected after 2 h (Fig. 6A, inset), but the mean particle height was increased to 5.1±0.2 nm. After 24 h from the start of aggregation, the globular oligomers were found to be assembled into clusters (Fig. 6B), while at 48 h bundles of fibrils, almost totally submerged by non fibrillar material, were observed (Fig. 6C and Fig.S6). Sample treatment with SDS and centrifugation, as detailed in Materials and Methods, were performed to recover the insoluble fibrillar fraction (Fig.6C, inset). Bundles of fibrils became the prevailing morphology observed after 72 h; treatment with SDS allowed us to better reveal the ultrastructure of these bundles. They turned out to be composed of fibrils about 4 nm high intertwined together and further assembled into bundles between 20 and 60 nm high (Fig. 6D).

## Discussion

This work was aimed at characterizing the structural features emerging during the aggregation in both normal (Q24) and expanded (Q55) AT3, as well as in the JD in isolation. We first monitored the kinetics of the process by ThT fluorimetry, which showed that all the variants investigated underwent aggregation, that AT3/182Δ (consisting of the sole JD) aggregated much more slowly than the two other forms, and that it displayed an evident lag phase, in keeping with the previously proposed nucleation-dependent mechanism [14]. This, of course, does not rule out that the two full-length variants also undergo a lag phase, which might be however undetectable due to the much higher aggregation rate. It is worth mentioning, in any case, that these results confirm the intrinsic amyloidogenic potential of the JD, as previously proposed [10], [11] but, at the same time, they highlight the effect of the C-terminal disordered domain to substantially enhance the rate of the process.

By dot-blot and filter trap assays we provided a further characterization of the intrinsic properties of these aggregates. In dot blots we used an antibody that is capable of selectively recognizing oligomers but not mature fibrils [20]; in filter trap assays an anti-AT3 antibody. Our results show that all three forms were initially recognized by the anti-oligomer antibody, but AT3/182Δ and AT3Q24 gave an approximately constant signal over time unlike AT3Q55, whose signal progressively faded and completely disappeared at a 30-h incubation (Fig. 1B). This was paralleled by the appearance of the filter-trap signal by the same expanded AT3 form after 30 h (Fig. 5), whereas in contrast, no reactivity was displayed by the two other variants.

To further confirm these findings we also characterized the morphology of the three AT3 variants by AFM, which actually showed that AT3Q55 underwent progressive aggregation, generating oligomers (Fig. 6A), which formed clusters after 24 h (Fig. 6B) and gave rise to bundles of tangled SDS-resistant fibrils at 48 and 72 h (Fig. 6C and D). Nevertheless, a closer inspection of the images also suggests that, taken individually, fibrils are unbranched. The morphology of these bundles of fibrils resembles that reported for synthetic polyQ peptides [34]. As expected, the normal full-length AT3Q24 only generated oligomers and short protofibrils even at the latest times of incubation (Fig. S5), and the JD generated a prevailing globular morphology (Fig. S4). These are among the first AFM images taken of AT3 during aggregation.

Overall, these results provide a comprehensive framework of the events taking place during aggregation of normal and expanded AT3 that confirms the previously proposed two-step kinetics [12], [13]. Nevertheless, very little is known so far regarding the structural changes accompanying the different stages of the process. We thus addressed this issue by taking advantage of FTIR spectroscopy. We collected second derivative spectra in the amide I band region of the three forms, before and during the incubation. Before the incubation they were fairly similar, indicative of α-helical/disordered structures (1657 cm^−1^ band), intramolecular β-sheets (1635 cm^−1^) and β-turns (1688 cm^−1^ and 1678 cm^−1^). The most notable difference was that the 1657 cm^−1^ band was more pronounced than the one around 1635 cm^−1^ in the full-length AT3 forms compared to the JD. This implies that the C-terminal disordered domain must contribute to the absorption of the 1657 cm^−1^ component, and that it is mainly α-helical and/or disordered, consistent with CD spectra and computational studies [11].

During the incubation, JD and AT3Q24 spectra underwent similar changes that point to a decrease in intramolecular β-sheet and α-helical/disordered structures, and a concurrent increase in intermolecular β-sheet, clearly representative of the aggregation process. However, the full-length variant aggregated much faster than the JD and, in addition, displayed a downshift of the β-intermolecular band from 1630 cm^−1^ to 1624 cm^−1^, suggestive of stronger interactions and/or increased number of β-strands. This clearly shows that the C-terminal domain also affects stability and structure of the aggregates, irrespective of whether it carries normal or expanded polyQs.

Although the aforementioned results make a significant contribution to the understanding of the mechanisms of AT3 fibrillogenesis, the key discovery of the present work is the elucidation of a major, distinctive structural feature arising during the aggregation of the expanded form but not of the normal one, namely hydrogen bonds among glutamine side-chains. Actually, by H/D exchange we could unambiguously assign the 1657 cm^−1^ and 1604 cm^−1^ bands to glutamines involved in strong side-chain-side chain (and possibly side chain-backbone) hydrogen bonding in the AT3Q55 mature amyloid aggregates. Therefore, based on our results, these are the hallmarks of mature fibrils generated by expanded AT3 variants. So they are associated with, and responsible for irreversible aggregation and SDS-insolubility of the expanded forms. As a matter of fact, the appearance of the side-chain glutamine signal after about 50 h of incubation was paralleled by the formation of irreversibly aggregated proteins and bundles of SDS-resistant, mature fibrils, as shown in filter trap assays and in AFM, respectively. Even more remarkably, the time course of the appearance of main-chain and side-chain hydrogen bonding showed that the former started from the very beginning of incubation, whereas the latter became significant after 30 h (Fig.6). So it is quite likely that the constraints generated at the level of the main chain are a prerequisite for side-chain hydrogen bonding, probably favoring the latter interaction entropically. Once either interaction is formed, the protein would remain locked in an irreversibly aggregated conformation.

Based on x-ray crystallography of a small glutamine peptide, i.e. D_2_Q_15_K_2_, and on theoretical considerations, Perutz and coworkers proposed that polyQs generate amyloid fibers consisting of at least two β-helical turns each of 20 residues, and that residues in successive turns would be linked by hydrogen bonds between both main chain and side chain amides [18]. After inspecting the same crystallographic data, Sikorski and Atkins [19] suggested an alternative model, in which the peptide adopts a hairpin conformation but still displays a similar pattern of hydrogen bonding.

Whatever the real structure of polyQ amyloids may be, these results highlight glutamine side-chain hydrogen bonding as a key structural feature for the generation of irreversible aggregates. At first glance, however, it is surprising that the models described rely upon experimental data obtained from a short polyQ peptide, whereas our observations unambiguously show that only expanded polyQ stretches can give rise to such aggregates and the related pattern of hydrogen bonding. These apparently conflicting results can be reconciled if one assumes that the protein context, in particular the flanking regions, play a crucial role in determining the aggregation pathway. In other words, even a short polyQ stretch in isolation would be subject to irreversible aggregation, whereas a much longer polyQ stretch would be required to achieve aggregation of normal sized proteins. Actually, the typical threshold for aggregation and onset of polyQ diseases is around 40 consecutive glutamines [35]. Interestingly, a recent paper shows that, in keeping with this hypothesis, synthetic polyQ peptides of non-pathological length (e.g., KKQ_32_KK) are much more prone to aggregation than a huntingtin exon 1 variant containing a Q_53_ stretch [36]. These results, along with ours and those of Perutz and Sikorski, substantiate the idea that glutamine side-chain hydrogen bonding is a common feature of all natural, irreversibly aggregated polyQ proteins.

As a final remark, it is worth mentioning that the FTIR spectroscopic methods adopted in the present work also allow one to monitor the progress of aggregation *in vivo* by detecting the FTIR signal in several model organisms, such as *E. coli* [37], yeast [38] and *Caenorhabditis elegans* [39], [40]. This provides an invaluable tool to correlate the emerging toxic effects in such organisms with the subtle structural features of the different aggregation products arising during the process.

## Materials and Methods

### Cloning, expression and purification of AT3 variants

Plasmid encoding the fragment 1–182 (that consists of the sole JD) was obtained by inserting, into the wild-type human AT3 (AT3Q24-isoform 2UIMs) gene cloned in pGEX-6P-1 (GE Healthcare LifeSciences, Little Chalfont, England), two stop codons downstream of the triplet encoding the residue 182. The truncated form (AT3/182Δ) was expressed in the *E. coli* strain BL21-*CodonPlus*(DE3)-RIL (*E. coli* B F^−^ *ompT hsdS* (r_B_^−^ m_B_^−^) *dcm* ^+^ Tet^r^ *gal*λ(DE3) *endA* Hte [*argU ileY leuW* Cam^r^] (Stratagene, La Jolla, CA, USA) in fusion with GST, with a Prescission Protease cleavage site in between, and purified as previously described [41]. The cDNA-encoding human wild type AT3Q24 was removed by *BamHI-SmaI* digestion from pGEX-6P-1 and subcloned in the pQE-30 vector (Qiagen Hamburg GmbH, Hamburg, Germany). The expanded form (AT3Q55) was chemically synthesized by Eurofins MWG Operon (Ebersberg, Germany) and cloned in the pQE30 vector. The two variants were expressed in SG13009 (*E. coli* K12 Nal^s^, Str^S^, Rif^S^, Thi^−^, Lac^−^, Ara^+^, Gal^+^, Mtl^−^_,_ F^−^, RecA^+^, Uvr^+^, Lon^+^; Qiagen Hamburg GmbH, Hamburg, Germany) as His-tagged proteins. The proteins were purified following the procedure reported by Chow et al. [42], except that only AT3Q55 was subjected to the last purification step, consisting of a gel filtration on a HiPrep 16/60 Sephacryl S-100 High Resolution column (GE Healthcare, Life Sciences, Little Chalfont, England), pre-equilibrated with PBS-G (20 mM potassium phosphate, pH 7.2, 150 mM NaCl, 1 mM 2-mercaptoethanol and 10% glycerol). Elution was performed at a flow rate of 0.5 ml/min in the same buffer. Fractions (1 ml) were collected and analyzed by SDS-PAGE electrophoresis. Fractions containing pure AT3Q55 were concentrated in an Amicon Ultra 30 K microconcentrator (Millipore, Bedford, MA, USA) and stored at −20°C. Before use, AT-3 variants were centrifuged at 15000×*g* for 15 min at 4°C to eliminate aggregates.

### Thioflavin T (ThT) assays

AT3 variants were incubated at a 25 µM concentration in PBS (25 mM potassium phosphate, pH 7.2, 0.15 M NaCl) at 37°C with 20 µM ThT, in clear-bottomed black Isoplate-96F/50B plates (Perkin Elmer, MA, USA) and read in a VICTOR TM X3 Multilabel Plate Reader (Perkin Elmer, MA, USA). Excitation and emission wavelengths were 445 nm and 535 nm, respectively. Readings were carried out from the bottom of the plates with no shaking and recorded every 30 min. Plates were sealed to prevent evaporation. Storage buffer was replaced by PBS immediately prior to each experiment using PD10 desalting columns (GE Healthcare LifeSciences, Little Chalfont, England). Protein content was determined using Comassie brilliant blue G-250 from Pierce (Pierce Biotechnology, Rockford, IL) and bovine serum albumin as a standard protein.

### Dot Blot and filter trap assays

Dot blot and filter trap assays were performed on AT3 samples incubated in PBS buffer at 37°C and a 1 mg/ml concentration without shaking. Dot blotting was performed by applying protein aliquots (13 µg) to a nitrocellulose membrane mounted on a manifold. Samples were vacuum-filtered and washed with 200 µl of PBS. Membranes were incubated in blocking solution (5% skim milk in PBS) for 60 min at room temperature and then probed for 60 min at room temperature with anti-amyloid oligomer (Abeta) antibody (Millipore, Bedford, MA, USA; Kayed et al., 2003) at 1∶5000 dilution in blocking solution. After incubation in primary antibody, membranes were washed thrice in 0.3% Tween in PBS for 10 min each time, and subsequently incubated for 60 min at 25°C in secondary antibody (horseradish peroxidase-goat anti-rabbit antibodies) at 1∶5000 dilution. Immunoreactive bands were revealed using ECL Western blotting reagent (GE Healthcare LifeSciences, Little Chalfont, England). For the filter trap assay, AT3 aliquots (10 µl-samples, 1 mg/ml protein concentration) were mixed with 500 µl of SDS buffer (50 mM Tris-HCl, pH 8.0, 5% SDS, 10 mM DTT, 100 mM NaCl, 1 mM EDTA). After a 10 min incubation at 100°C, the resulting samples were applied to a cellulose acetate membrane (0.2 µm pore size) mounted on a manifold, vacuum-filtered, washed once with 100 µl of SDS buffer and finally with 200 µl of PBS buffer. SDS-stable aggregates retained on the membrane were detected by immunoblotting analysis using anti-human AT3Q26 Z46 polyclonal antibody [41]. Membranes were incubated for 60 min at room temperature with the antibody at 1∶5000 dilution in blocking solution. After incubation in primary antibody, membranes were rinsed thrice in 0.3% Tween in PBS for 10 min each time, and incubated for 60 min at 25°C in secondary antibody (horseradish peroxidase-goat anti-rabbit antibodies) at 1.5000 dilution. Immunoreactive bands were revealed using ECL Western blotting reagent (GE Healthcare LifeSciences, Little Chalfont, England).

### FTIR spectroscopy

For the FTIR study of the AT3 variants, measurements were performed in attenuated total reflection (ATR) on a single reflection diamond element (Golden Gate, USA). An aliquot of about 5-10 µl of the protein solution (in PBS at a concentration of 1 mg/ml) was deposited on the diamond ATR plate and dried at room temperature in order to obtain a protein hydrated film. Its ATR/FTIR spectrum was then measured using the FTS40-A spectrometer (Digilab, USA), equipped with a nitrogen cooled Mercury Cadmium Telluride (MCT) detector and carefully purged by dry air to avoid interference with water vapor under the following conditions: 2 cm^−1^ spectral resolution, 20 kHz scan speed, 1000 scan co-additions, triangular apodization. Second derivatives of the spectra were obtained by the Savitzky-Golay algorithm (5 points), after an 11 point binomial smoothing of the measured spectra, using the software Grams/AI (Thermogalactic, USA). To study the aggregation kinetics, the three AT3 variants were incubated at 37°C in PBS, and the ATR/FTIR spectra of their hydrated films were measured at different times. For the hydrogen/deuterium exchange experiments, two different procedures were employed: i) the freshly prepared AT3Q55 variant was resuspended in heavy water and incubated at 37°C for up to 168 h. Under these conditions, H/D exchange took place during the protein misfolding and aggregation. In this way, deuterated aggregates were obtained and measured in ATR/FTIR as described above; ii) AT3Q55 mature aggregates were obtained after incubation of the native protein in H_2_O/PBS at 37°C for 168 h. To remove and replace H_2_O with D_2_O, aggregates were subjected to, three cycles of lyophilization and resuspension in D_2_O. After each resuspension, they were incubated at room temperature for 3 h. The final sample was again resuspended in D_2_O and incubated at room temperature for 17 h before ATR/FTIR measurements.

### AFM

AT3 was incubated at a 1 mg/ml-concentration in PBS at 37°C. Samples for AFM inspection were prepared at fixed aggregation times using two different procedures. In the first procedure, a 10 µl aliquot was withdrawn, incubated on a freshly cleaved mica substrate for 5 min, then rinsed with Milli-Q water and dried under mild vacuum. In the second procedure, a 2 µl aliquot was diluted 400-fold and 10 µl of the diluted solution were deposited on mica and then dried under mild vacuum. When imaged by AFM, samples prepared according these two procedures displayed similar features, only differing in the aggregate density. To eliminate the non-fibrillar material masking AT3Q55 fibril morphology at long aggregation times (48 and 72 h), and to recover the insoluble fibrillar fraction, 30 µl aliquots of AT3Q55 were added to 300 µl of SDS buffer (50 mM Tris-HCl, pH 8.0, 5% SDS, 10 mM DTT, 100 mM NaCl, 1 mM EDTA) and boiled at 100°C for 10 min. The sample was then centrifuged at 15000×g for 15 min and the supernatant was replaced by Milli-Q water; this centrifugation and washing procedure was repeated three times in total. The pellet was finally suspended in Milli-Q water. For AFM imaging, the fibril suspension was diluted 100-fold and 10 µl of the diluted sample were deposited on mica and then dried under mild vacuum. AFM images were acquired in tapping mode in air using a Dimension 3100 Scanning Probe Microscope equipped with a ‘G’ scanning head (maximum scan size 100 µm) and driven by a Nanoscope IIIa controller, and a Multimode Scanning Probe Microscope equipped with “E” scanning head (maximum scan size 10 µm) and driven by a Nanoscope IV controller (Digital Instruments - Veeco, Santa Barbara, CA). Single beam uncoated silicon cantilevers (type OMCL-AC160TS, Olympus, Tokyo, Japan) were used. The drive frequency was between 320 and 340 kHz, the scan rate was between 0.5 and 2.0 Hz. Aggregate heights were measured from the height in cross section of the topographic AFM images; standard errors are reported. Due to the drying procedure applied, the measured heights reported in the Results are reduced with respect to fully hydrated conditions. A shrinking factor of 2.2 has been evaluated by comparing the heights of a globular protein under liquid and in air after drying under vacuum [43].

## Supporting Information

Figure S1 **FTIR second derivative spectra of AT3 variant aggregates.** AT3 variants were incubated at 37°C for 168 h. The spectra of the pelletted aggregates are reported after normalization at the tyrosine peak.
(TIF)

Figure S2 **FTIR absorption spectra of freshly purified (blue profile) and mature pelleted aggregates (red profile) of AT3 variants.**
(TIF)

Figure S3 **FTIR spectra of AT3Q55 aggregates treated with SDS buffer.**
(TIF)

Figure S4 **Tapping mode AFM images (height data) of the truncated variant AT3/182Δ.** The protein was incubated at 37°C for: (A) 0 h; (B) 24 h; (C) 72 h. Scan size 2 µm, Z range (A) 10 nm; (B) 20 nm; (C) 15 nm.
(TIF)

Figure S5 **Tapping mode AFM images (height data) of the normal full length variant AT3Q24.** The protein was incubated at 37°C for: (A) 0 h; (B) 24 h; (C) 72 h. Scan size 2 µm, Z range 20 nm.
(TIF)

Figure S6 **Tapping mode AFM image (left, height data; right, amplitude data) of the expanded variant AT3Q55.** The protein was incubated at 37°C for 48 h. Non fibrillar material completely covering a fibril bundle is apparent in the middle of the image. A portion of another bundle is on the left. This unstructured material can be removed by sample treatment with SDS, to reveal the fibril morphology (see Fig.6). Scan size 1.9 µm, Z range 200 nm.
(TIF)
