# Supplementary figures and images for: A Major Role for Side-Chain Polyglutamine Hydrogen Bonding in Irreversible Ataxin-3 Aggregation
Source: PLoS One. 2011 Apr 13;6(4):e18789. doi: 10.1371/journal.pone.0018789 (PMC3076451; doi:10.1371/journal.pone.0018789)

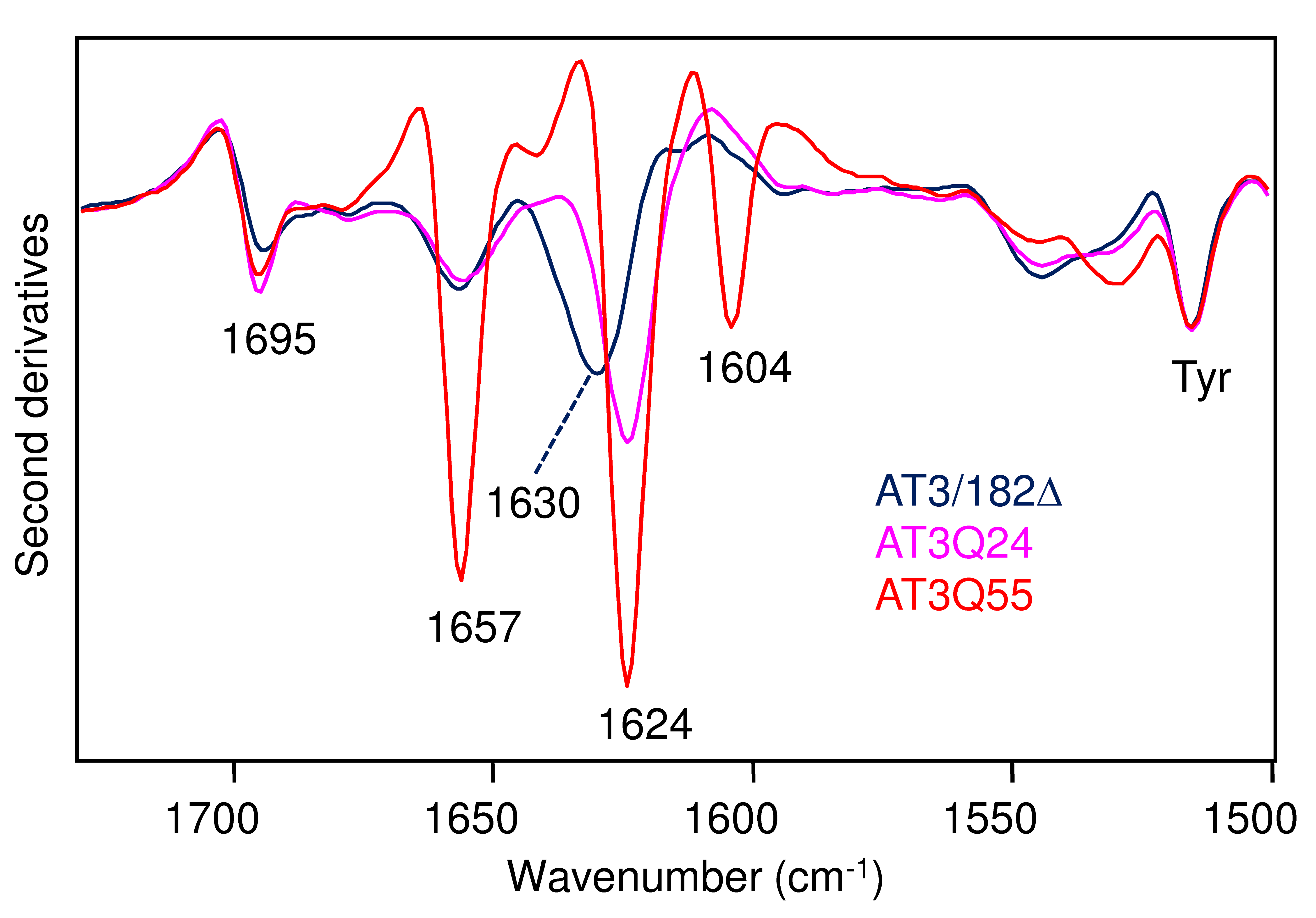

Supplement: Figure S1 — FTIR second derivative spectra of AT3 variant aggregates. AT3 variants were incubated at 37°C for 168 h. The spectra of the pelletted aggregates are reported after normalization at the tyrosine peak. (TIF) [file pone.0018789.s001.tif]

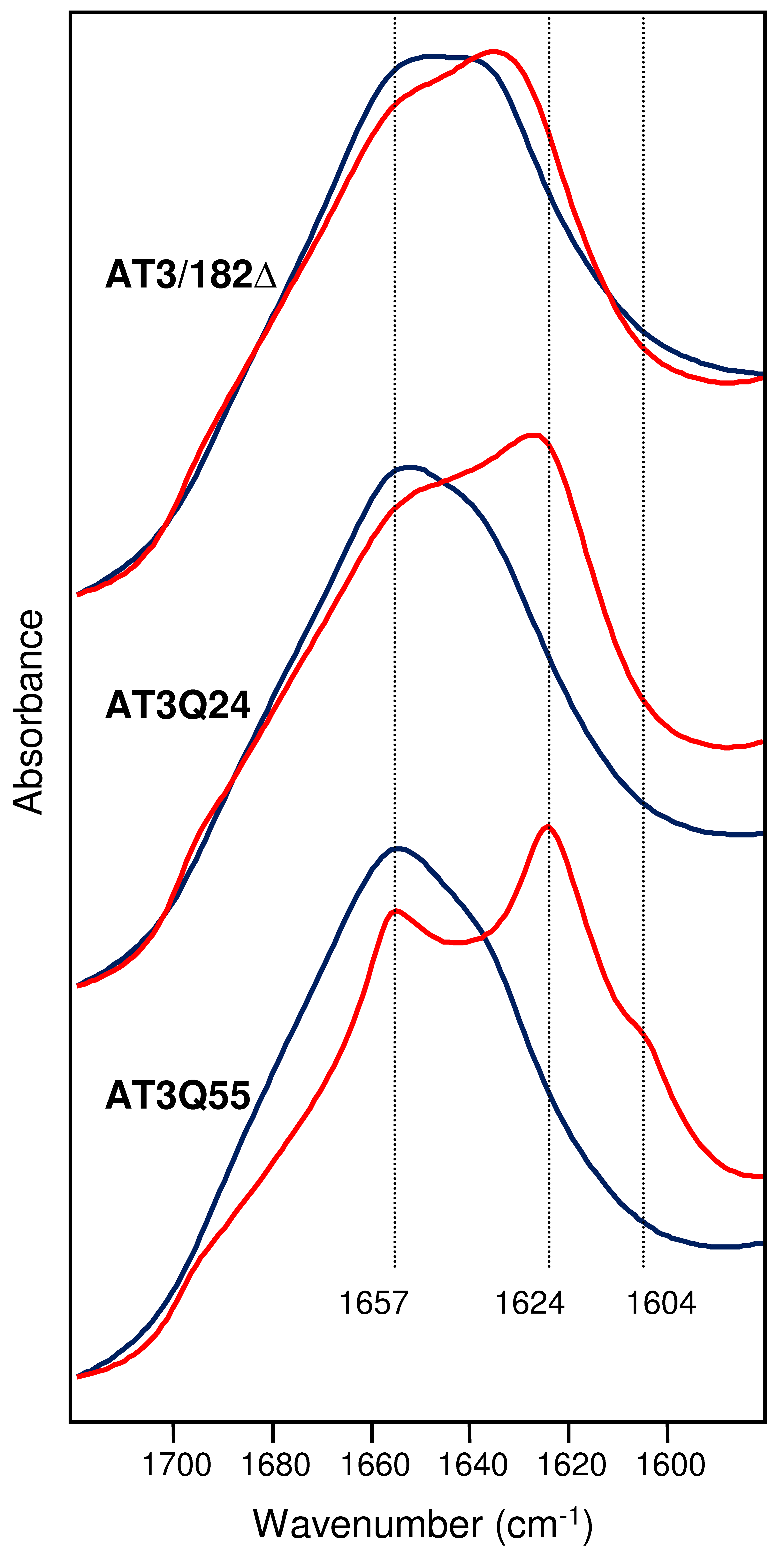

Supplement: Figure S2 — FTIR absorption spectra of freshly purified (blue profile) and mature pelleted aggregates (red profile) of AT3 variants. (TIF) [file pone.0018789.s002.tif]

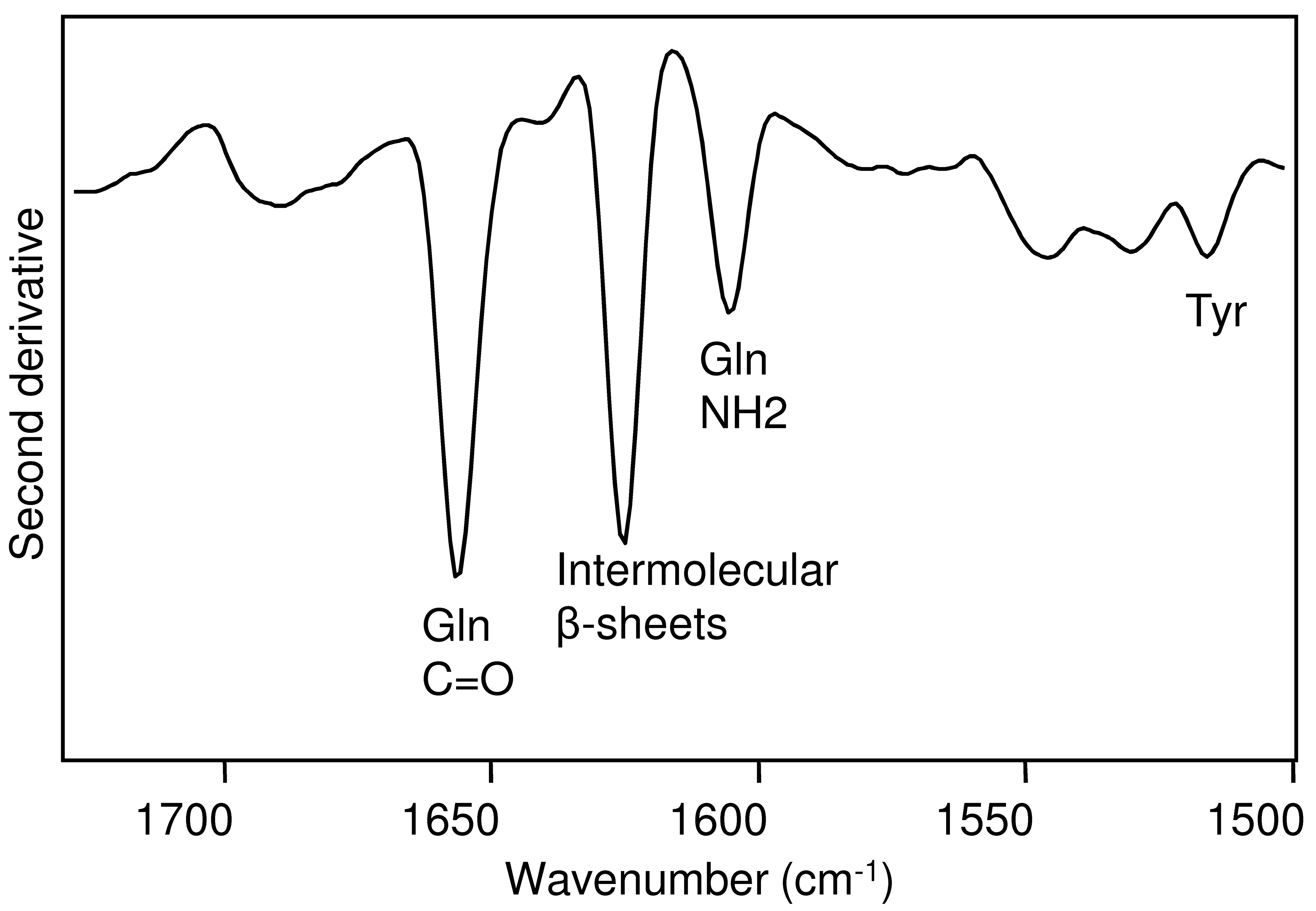

Supplement: Figure S3 — FTIR spectra of AT3Q55 aggregates treated with SDS buffer. (TIF) [file pone.0018789.s003.tif]

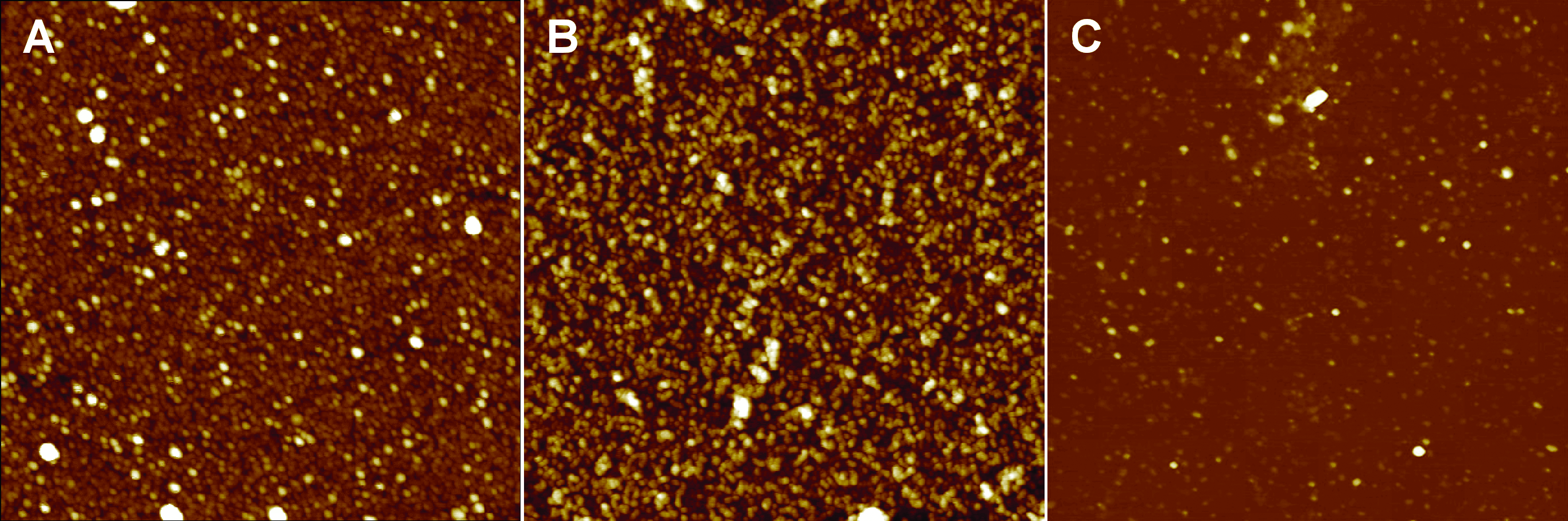

Supplement: Figure S4 — Tapping mode AFM images (height data) of the truncated variant AT3/182Δ. The protein was incubated at 37°C for: (A) 0 h; (B) 24 h; (C) 72 h. Scan size 2 µm, Z range (A) 10 nm; (B) 20 nm; (C) 15 nm. (TIF) [file pone.0018789.s004.tif]

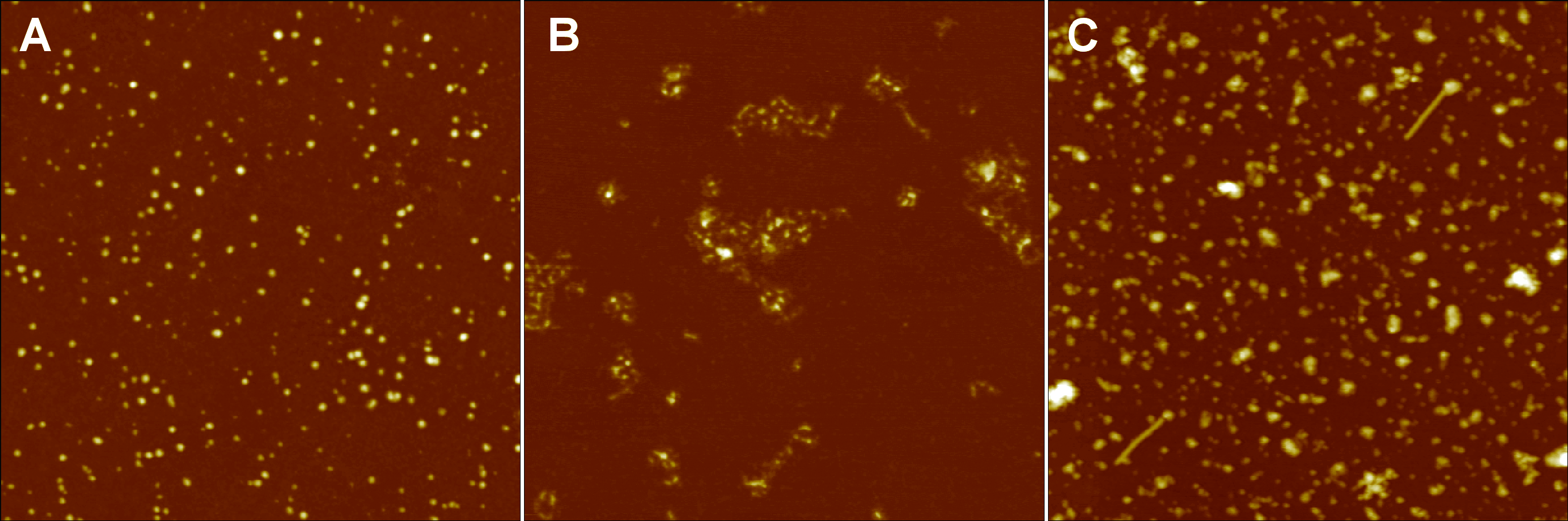

Supplement: Figure S5 — Tapping mode AFM images (height data) of the normal full length variant AT3Q24. The protein was incubated at 37°C for: (A) 0 h; (B) 24 h; (C) 72 h. Scan size 2 µm, Z range 20 nm. (TIF) [file pone.0018789.s005.tif]

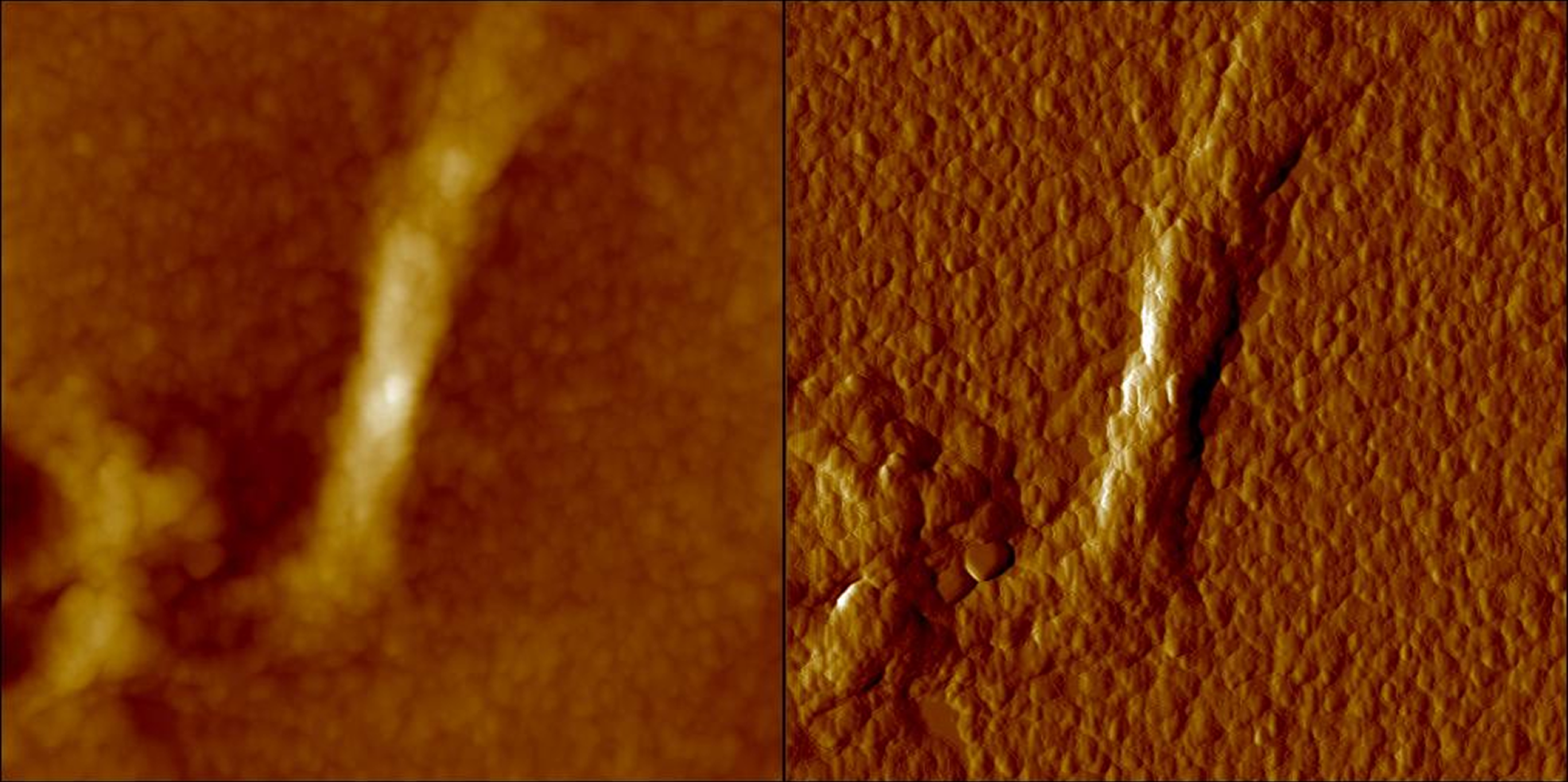

Supplement: Figure S6 — Tapping mode AFM image (left, height data; right, amplitude data) of the expanded variant AT3Q55. The protein was incubated at 37°C for 48 h. Non fibrillar material completely covering a fibril bundle is apparent in the middle of the image. A portion of another bundle is on the left. This unstructured material can be removed by sample treatment with SDS, to reveal the fibril morphology (see Fig.6). Scan size 1.9 µm, Z range 200 nm. (TIF) [file pone.0018789.s006.tif]
